# Performance Evaluation of Biochip Chemiluminescent Immunoassay for Screening Seven Mycotoxins in Wheat Flour Simultaneously

**DOI:** 10.5812/ijpr-140356

**Published:** 2023-12-06

**Authors:** Mahraz Osouli, Hassan Yazdanpanah, Jamshid Salamzadeh, Samira Eslamizad

**Affiliations:** 1 Food Safety Research Center, Shahid Beheshti University of Medical Sciences, Tehran, Iran

**Keywords:** Mycotoxins, Aflatoxin, Immunoassay, Simultaneous Screening, Cereal

## Abstract

**Background:**

Wheat grains are susceptible to mycotoxins, toxic natural secondary metabolites generated by certain fungi on agricultural produce in the field during growth, harvest, transportation, or storage. Therefore, wheat flour can be contaminated with mycotoxins, which seriously threaten human health.

**Methods:**

A rapid method for screening seven mycotoxins in wheat flour was validated in accordance with Commission Decision 2002/657/EC. With this multi-analytical screening method, 7 prevalent mycotoxins (fumonisin B1, ochratoxin A, aflatoxin G1, deoxynivalenol, T-2 toxin, aflatoxin B1, and zearalenone) can be determined simultaneously. The method’s applicability was demonstrated by screening 7 mycotoxins in 39 wheat flour samples collected from different bakeries in Tehran province, Iran.

**Results:**

The validation results indicated that for all 7 mycotoxins, the positivity threshold (T) was above the cut-off value (Fm), and no false positive results were obtained for any of the mycotoxins. The screening results of 12 packaged and 27 bulk wheat flour samples indicated that the concentrations of all mentioned mycotoxins were higher than the cut-off (in the relative light unit [RLU]), and all the samples were compliant.

**Conclusions:**

The present study revealed that the biochip-based technique is valid for identifying and assessing the levels of 7 mycotoxins in grain samples, such as wheat flour, at the measured validation concentrations. The method was simple, fast, and able to screen 7 mycotoxins simultaneously. The test process of the kit is easy to conduct, and the results are straightforward to interpret.

## 1. Background

Wheat (*Triticum aestivum* L.) belongs to the Poaceae grass family, and approximately one-third of the world’s grain production is dedicated to it (1). Wheat plays a crucial role in providing about four-fifths of the total daily caloric and protein intake in developing countries, making it a staple crop (2, 3). However, there is a significant risk of wheat grains becoming contaminated with mycotoxins when exposed to molds. Mycotoxins are secondary metabolites produced by certain species of fungi, and their toxic nature poses severe health risks, including carcinogenicity, hepatotoxicity, nephrotoxicity, and endocrine disruption (4, 5).

The Food and Agricultural Organization (FAO) of the United Nations has estimated that approximately one-quarter of the world’s crops are contaminated with mycotoxins each year, resulting in substantial economic losses and posing a significant threat to human health (6). Mycotoxins that are well-known for their dangers to human health can be categorized into *Aspergillus mycotoxins* (e.g., aflatoxins), *Penicillium mycotoxins* (e.g., ochratoxin A and citrinin), and *Fusarium mycotoxins* (e.g., zearalenone, deoxynivalenol, fumonisins, trichothecenes, nivalenol, T-2 toxin, beauvericin, and enniatin) (7).

Aflatoxins, for example, have diverse toxicological effects on populations due to various types of exposure, resulting in acute and chronic effects. Aflatoxins are known to be mutagenic, teratogenic, carcinogenic, and immunosuppressive toxins (8). Ochratoxin A has been classified as possibly carcinogenic to humans (group 2B) and can cross the placental barrier and be excreted in breast milk. Zearalenone, classified as group 3 (not classifiable as to its carcinogenicity to humans), is primarily known for its estrogenic effects (7). Deoxynivalenol, also categorized as group 3 (not classifiable as to its carcinogenicity to humans), has been observed to induce vomiting, anorexia, decreased weight gain, and impaired immune function in various animal species. Numerous cases of gastroenteritis in diverse populations worldwide suggest a possible connection to dietary exposure to deoxynivalenol (9).

The International Agency for Research on Cancer (IARC) classified fumonisin B1 as a group 2B possible carcinogen for humans. Fumonisin B1 has been associated with hepatocarcinoma, immune system stimulation and suppression, neural tube defects, nephrotoxicity, and various other health issues (10). T-2 toxin has the potential to affect cellular immune responses in animals and inhibit protein and deoxyribonucleic acid (DNA) synthesis. It has been linked to various toxicities in animals and humans, including alimentary toxic aleukia, Mseleni joint disease, scabby grain toxicosis, and Kashin-Beck disease (11).

The European Union (EU) and other international regulators have established maximum levels (MLs) for certain mycotoxins in food, including wheat, to safeguard consumer health from their harmful effects (12). These MLs necessitate the use of selective and sensitive analytical methods to detect very low concentrations of these metabolites in wheat flour samples. Specifically, the EU has set safe MLs for some mycotoxins in wheat samples, including 5, 4, 1250, 2, and 100 µg/kg for ochratoxin A, aflatoxin G1, deoxynivalenol, aflatoxin B1, and zearalenone, respectively (12). The determination of mycotoxins in cereals and related food products is a critical practice to ensure food safety (13). However, the analysis of mycotoxins in cereals poses challenges due to their typically low concentrations and the complexity of the matrices in these foods (14).

High-performance liquid chromatography (HPLC) coupled with various detectors, such as ultraviolet (UV), fluorescence, diode array, liquid chromatography-mass spectrometry (LC-MS), and liquid chromatography-tandem mass spectrometry (LC-MS/MS), has proven to be a powerful tool for the analysis and detection of important mycotoxins (15). These analytical methods provide relatively quick results when applied to food or feed samples, typically within hours or days. Most of these techniques involve solid-phase column cleanup of extracts and immunoaffinity methods to eliminate interferences and enhance the accuracy of mycotoxin measurement. The increasing complexity of analytical processes in the food industry calls for the prompt reporting of each individual contaminant. Moreover, competition within the food and feed industry demands cost reduction, the utilization of cheaper labor, and the swift delivery of goods. As a result, rapid methods for mycotoxin analysis have gained increasing importance (16).

Immunoassay-based methods, such as enzyme-linked immunosorbent assay (ELISA) and lateral flow devices (LFDs), play a crucial role in the rapid analysis of mycotoxins (15). Biochip array technology, a type of immunoassay-based technology, enables the semi-quantitative simultaneous determination of multiple analytes in samples using miniaturized immunoassays conducted on a semi-automated analyzer known as the Evidence Investigator (17). Biochip array technology enhances result throughput and offers significant cost-effectiveness advantages for mycotoxin screening (17). The testing procedure for the Myco 7 kit is user-friendly, and the generated results are easily interpretable (18). This method is relatively fast and allows for the simultaneous testing of mycotoxins.

## 2. Objectives

This article presents the results of the evaluation and validation of the Myco 7 microarray kit using various wheat flour samples to assess compliance with the detection limits specified by the manufacturer. The validation of this kit has been conducted in accordance with European Commission Decision No. EC/2002/657 (19). The validated method was applied to 39 bulk wheat flour samples and packaged wheat flour samples collected from bakeries and markets in Tehran province, Iran, for the first time.

## 3. Methods

### 3.1. Standards and Apparatus

Fumonisin B1 (FUM), ochratoxin A (OTA), aflatoxin G1 (AFG1), deoxynivalenol (DON), T-2 toxin (T2), aflatoxin B1 (AFB1), and zearalenone (ZEA) were obtained from Supelco Incorporation (USA). Myco 7 kit (EV 4065) was purchased from Randox Company (UK).

Vortex (Heidolph, Germany) model Hei-MIX Reax top, roller mixer (Behdad, Iran) model BMW-4-1-10-R-1-89, centrifuge (Hettich, Germany) Rotinta 380R and Evidence Investigator (Randox Food Diagnostics, UK) were used.

### 3.2. Blank and Real Wheat Flour Samples

Twenty blank wheat flour samples were collected from various bakeries. These samples of wheat flour underwent analysis to confirm the absence of all 7 mycotoxins (FUM, OTA, AFG1, DON, T2, AFB1, and ZEA). Additionally, 20 spiked samples were prepared by introducing the working standard solution into blank wheat flour at varying spiking levels. Furthermore, 39 real wheat flour samples were procured from Tehran province, spanning the period within April 2017 to March 2018. These samples were stored at -20°C before being prepared for analysis.

### 3.3. Preparing of Standard Solutions

Standard stock solutions of each mycotoxin were prepared at a concentration of 200,000 ng/g in methanol, except for fumonisin B1, which was dissolved in a mixture of deionized water and methanol (50:50) at a concentration of 100,000 ng/g. An intermediate standard solution containing 10,000 ng/g of ochratoxin A, aflatoxin G1, T-2 toxin, and aflatoxin B1 in methanol was also prepared.

### 3.4. Biochip Array Technology

The Myco 7 (EV 4065) is a commercial kit that simultaneously quantitatively tests for mycotoxins. Each kit contains 6 carriers, with each biochip carrier comprising 9 wells coated with immobilized specific antibodies. The antibodies vary depending on the type of microarray kit; the Microarray Myco 7 is specific for 7 mycotoxins.

### 3.5. Sample Preparation

First, 5 grams of each sample was homogenized and weighed. Afterward, 25 mL of a mixture of acetonitrile, methanol, and water (50:40:10) was added to the samples. The mixtures were then vortexed for 60 seconds and rolled for 10 minutes, followed by centrifugation at 3000 rpm for 10 minutes. For dilution in ready-to-use wash buffer, 50 µL of samples and 150 µL of wash buffer were mixed. Finally, 150 µL of diluted wash buffer and 50 µL of each sample or calibrator were added to each well, respectively.

### 3.6. Assay Protocol for Cereal Samples

Following the manufacturer’s instructions, 150 µL of diluted wash buffer and 50 µL of calibrator/control/samples were added to each well, respectively. The handling tray was lightly tapped to ensure proper mixing of the reagents. The holding tray was incubated in a thermal shaker for 30 minutes at 25°C and 370 rpm. The conjugate solution was prepared by adding 1 mL of deionized water and rolling it for 15 minutes. Afterward, 100 µL of ready-to-use conjugate was added to each well of the biochip and incubated for 60 minutes at 25°C and 370 rpm. After this step, the handling tray was immediately subjected to 2 quick wash cycles followed by an additional four wash cycles, each lasting 2 minutes, using a diluted wash buffer. Finally, any residual wash buffer was removed using a lint-free tissue. Then, 250 µL of working signal reagent was added to each biochip well to cover and protect it from light. After exactly 2 minutes (± 10 seconds), the carrier was placed in the Evidence Investigator, and images were captured using dedicated software.

### 3.7. Evidence Investigator Analyzer

The technology of the Evidence Investigator^TM^ is based on a solid substrate called a biochip. The identification technology in this device relies on a competitive chemiluminescent immunoassay. Higher levels of mycotoxins in the samples lead to less binding of horseradish peroxidase (HRP)-labeled mycotoxin, which can result in reduced emission of chemiluminescent signals recorded by a charged-coupled device (CCD) camera and presented in the relative light unit (RLU) and part per billion (ppb). The concentration of unknown samples is measured using the calibration curve.

### 3.8. Image and Data Handling

The detection of biochips is based on a chemiluminescence signal recorded by a CCD camera. The CCD camera captures all the light emissions emitted during the chemical reaction in each biochip well. The signals are then translated into concentration and RLUs using image processing software.

### 3.9. Validation Procedure

The validation was performed on the basis of the European Decision 2002/657/EC (19). Specificity, detection capability (CCβ), practicability, applicability, and stability are the success factors that have been identified.

### 3.10. Number of Samples for Validation

To validate screening methods, European guidelines specify that the screening target concentration should be set at a level equal to or below half of the regulatory/action limit, denoted as ½ maximum residue limit (MRL). This requirement ensures that, in order to demonstrate that the CCβ is within the regulatory limit and equal to or below ½ MRL, only one or no incorrectly compliant result should occur after testing a minimum of 20 positive screening control samples (19).

### 3.11. Determination of Cut-Off Level and CCβ

The validation of screening methods, whether qualitative or semi-quantitative, all require a cut-off value to identify samples at or above those screening positive and subject to physicochemical confirmation (20). Cut-off levels and CCβ were calculated for the seven mycotoxins (fumonisin B1, ochratoxin A, aflatoxin G1, deoxynivalenol, T-2 toxin, aflatoxin B1, and zearalenone). This study calculated the signal in RLU; this study has calculated the signal in RLU. The mean and standard deviation of spiked and blank signals in RLU were calculated for each mycotoxin (Table 1). Eventually, the threshold value T is calculated from the blanks as follows:

T = Average signal of blank in RLU - 1.64×SD signal of blank in RLU

In addition, the cut-off factor (Fm) is calculated from the spiked samples as follows:

Fm = Average signal of spiked in RLU + 1.64 × SD signal of spiked in RLU

During validation, if the threshold exceeds Fm, the detection level (CCβ) is equivalent to the target concentration; however, if Fm is greater, the concentration of mycotoxins in the validation can be increased.

**Table 1.** Calibration Range, Limit of Detection (LOD), Maximum Level (ML) in European Commission (EC) and Iran (12, 21), and Spiking Level of Mycotoxins

Mycotoxins	Calibration Range, ppb	LOD, ppb	ML, According to EC, µg/kg	ML in Iran, µg/kg	Selected Spike Level, ppb
**FUM**	0-80	10	-	-	30
**OTA**	0-16	0.25	5.0	5.0	5
**AFG1**	0 - 20	0.5	4.0	-	2
**DON**	0 - 2000	100	1250	1000	1250
**T2**	0 - 80	5	-	-	25
**AFB1**	0 - 2.54	0.25	2	5	2
**ZEA**	0 - 40	2.5	100	200	36

Abbreviations: FUM, fumonisin B1; OTA, ochratoxin A; AFG1, aflatoxin G1; DON, deoxynivalenol; T2, T-2 toxin; AFB1, aflatoxin B1; ZEA, zearalenone; LOD, limit of detection; ML, maximum level.

### 3.12. Practicability

A feasibility study is not a separate test that would require additional testing. The practicability study aimed to determine whether the approach is suitable for everyday practice or not. Since the validation, the analysts have checked the practicability by assessing the ease of the method under normal conditions. Essential equipment (special or common equipment in a laboratory), instruments (specific or common tools in a laboratory), reagents (ready to use or not), and environmental conditions were evaluated.

### 3.13. Applicability

Miscellaneous samples of packaged and bulk wheat flour, which is used for making various confectionery products and traditional breads, were chosen to illustrate different wheat flour groups. The kit’s applicability for different wheat flour varieties was tested by assessing the CCβ of the 7 mycotoxins from 39 different samples.

### 3.14. Application of the Method

All 39 bulk and packaged wheat flour samples collected from different areas of Tehran province were analyzed simultaneously using the validated screening method to detect the presence of 7 mycotoxins and confirm the method’s suitability and capability.

## 4. Results

### 4.1. Detection Capabilities

**Figure 1.**
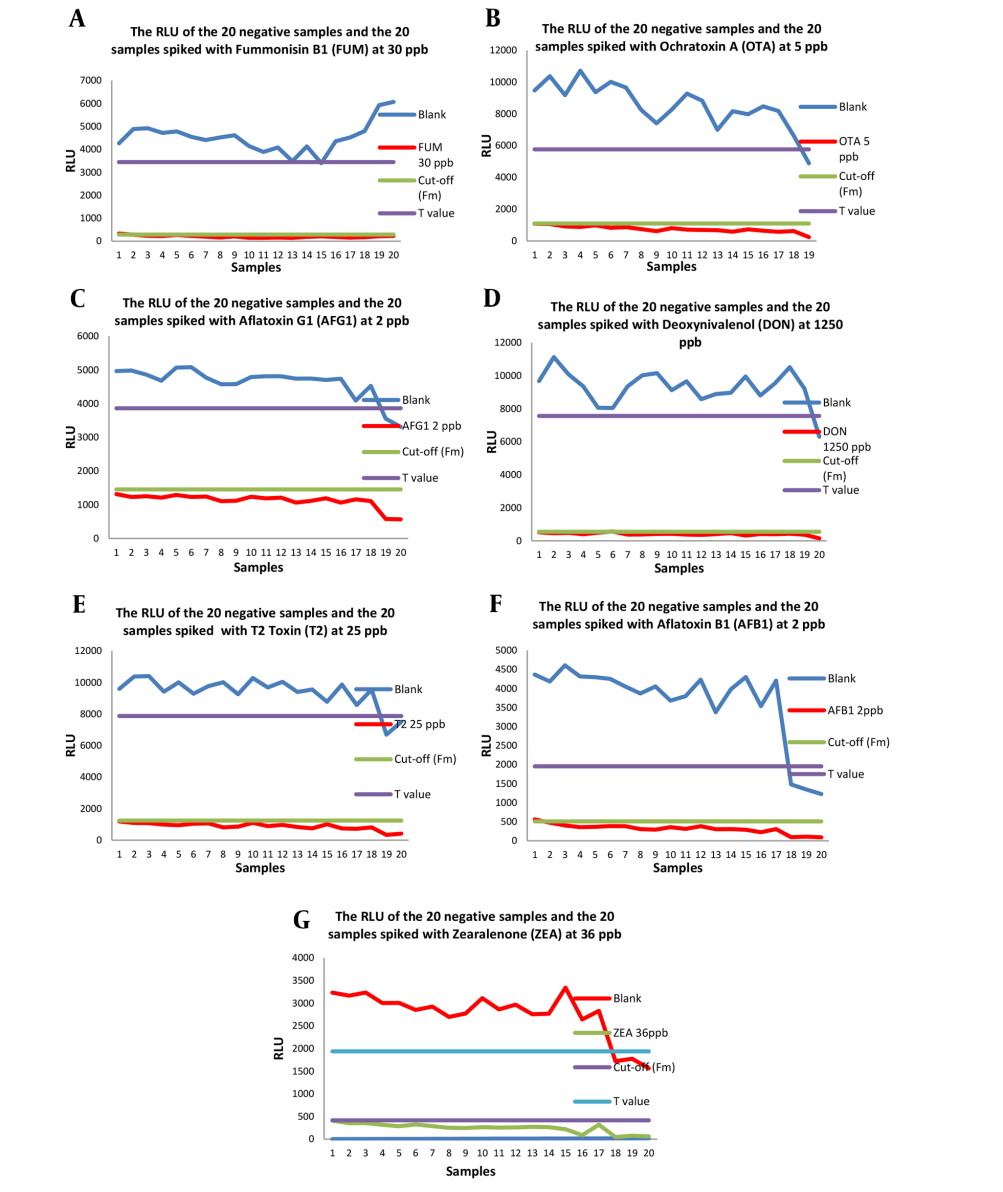
Relative light unit (RLU) of the 20 blank and the 20 Spiked Samples for 7 mycotoxins, A, fumonisin B1 (FUM), B, ochratoxin A (OTA), C, aflatoxin G1 (AFG1), D, deoxynivalenol (DON), E, T-2 Toxin (T2), F, aflatoxin B1 (AFB1), and G, zearalenone (ZEA)

The scatterplots of the results for the 20 spiked samples containing seven mycotoxins and the 20 negative samples are displayed in Figure 1. The summarized records when Fm is selected as the cut-off value are presented in Table 2, as T in RLU was higher than Fm for all mycotoxins. No more than 5% of false negative results were obtained for all seven mycotoxins, demonstrating that the results are acceptable and the validated concentration corresponds to CCβ. The screening target concentration for approved analytes is at or below the regulatory limit (MRL), and the selected spike values (validation concentration) were chosen as CCβ according to Commission Decision 2002/657/EC (Table 3) (19).

**Table 2.** False Positive and False Negative Rates of Biochip Chemiluminescent Immunoassay for Different Mycotoxins When Fm Is Taken as a Cut-off Value

Parameters	FUM	OTA	AFG1	DON	T2	AFB1	ZEA
**Concentration, µg/kg**	30	5	2	1250	25	2	36
**T value, RLU**	3448.03	5765.24	3862.01	7561.01	7873.37	1956.05	1938.19
**Fm value, RLU**	286.53	1099.82	1454.95	549.71	1243.15	510.04	416.10
**T > Fm**	Yes	Yes	Yes	Yes	Yes	Yes	Yes
**Number of false positive**	0	0	0	0	0	0	0
**False positive rate, %**	0	0	0	0	0	0	0
**Number of false negatives**	1	0	0	1	0	1	0
**False negative rate, %**	5	0	0	5	0	5	0

Abbreviations: RLU, Relative light Unit; FUM, fumonisin B1; OTA, ochratoxin A; AFG1, aflatoxin G1; DON, deoxynivalenol; T2, T-2 toxin; AFB1, aflatoxin B1; ZEA, zearalenone.

**Table 3.** Results of Detection Capabilities (CCβ)

Parameters	FUM	OTA	AFG1	DON	T2	AFB1	ZEA
**Announced LOD Randox, µg/kg**	10	0.25	0.5	100	5	0.25	2.50
**Spike level used for validation, µg/kg**	30	5	2	1250	25	2	36
**CCβ, µg/kg**	30	5	2	1250	25	2	36

Abbreviations: FUM, fumonisin B1; OTA, ochratoxin A; AFG1, aflatoxin G1; DON, deoxynivalenol; T2, T-2 toxin; AFB1, aflatoxin B1; ZEA, zearalenone; LOD; Limit of Detection.

### 4.2. Practicability

Only a small portion of the wheat flour sample was required (5 g). Minimal sample pre-treatment is required for the samples. The kit contained sufficient material, and the analysis was easy to perform. The software was easy to use and functional. The results were presented in RLU and ppb.

### 4.3. Specificity and False Positive Rate

Twenty negative flour samples and 20 spiked wheat flour samples were examined within 3 days for a validation study of the method. As the results are presented in Table 4, when T was selected as the cut-off, at least one sample or more out of 20 was screened false positive for all seven mycotoxins, and no false negative results were observed for them. When Fm was chosen as the cut-off, one sample out of 20 (5%) was falsely negative for FUM, DON, and AFB1, and no falsely positive results were obtained. Although choosing T as the cut-off value provided higher sensitivity due to the lack of false negative results, the false positive results led to expensive confirmatory analyses. On the other hand, choosing Fm as the cut-off level could strike a balance between sensitivity and the lack of a confirmatory test requirement. Consequently, Fm was selected as the cut-off point to determine the positivity of a real sample.

**Table 4.** False Positive and False Negative Results Regarding the Choice of the Threshold T or the Fm as Cut-off Level

Parameters	FUM	OTA	AFG1	DON	T2	AFB1	ZEA
**T (n = 20)**	3448.03	5765.24	3862.01	7561.01	7873.37	1956.05	1938.19
**Cut off = T (n = 20)**							
False positive	1	2	2	1	2	3	3
False negative	0	0	0	0	0	0	0
**Fm (n = 20)**	286.53	1099.82	1454.95	549.71	1243.15	510.04	416.10
**Cut off = Fm (n = 20)**							
False positive	0	0	0	0	0	0	0
False negative	1	0	0	1	0	1	0

Abbreviations: FUM, Fumonisin B1; OTA, Ochratoxin A; AFG1, Aflatoxin G1; DON, Deoxynivalenol; T2, T-2 Toxin; AFB1, Aflatoxin B1, ZEA, Zearalenone.

### 4.4. Applicability

The specificity study and the determination of CCβ in different types of wheat flour samples proved the applicability of the Myco 7 kit. The type of packaging of wheat flour samples did not affect the reliability of the results. This Myco 7 kit is suitable for a variety of cereals.

### 4.5. Stability of Mycotoxins

The stability of mycotoxins has been established through some literature reviews. Methanol standard solutions of fumonisin B1, ochratoxin A, deoxynivalenol, zearalenone, aflatoxin G1, and aflatoxin B1 stored at -18°C were stable for at least 14 months (22, 23). Moreover, all the Myco 7 biochip array kit components were observed to be stable for 2 years (17).

### 4.6. Analyses of Flour Samples

The mentioned method was used for screening 7 validated mycotoxins in real bulk and packaged wheat flour samples. The samples with RLUs higher than the cut-off were flagged as probable negative. The samples with RLUs below the cut-off level were listed as screening positive. Table 5 shows the data from screening bulk and packaged wheat flour samples.

**Table 5.** Incidence of 7 Mycotoxins in Bulk and Packaged Wheat Flour Samples in Relative Light Unit (RLU)

Parameters	FUM	OTA	AFG1	DON	T2	AFB1	ZEA
**Number of samples**	39	39	39	39	39	39	39
**Cut-off**	286.53	1099.82	1454.95	549.71	1243.15	510.04	416.10
**Number of positive sample**	0	0	0	0	0	0	0
**Positive sample (%)**	0	0	0	0	0	0	0

Abbreviations: FUM, fumonisin B1; OTA, ochratoxin A; AFG1, aflatoxin G1; DON, deoxynivalenol; T2, T-2 toxin; AFB1, aflatoxin B1; ZEA, zearalenone.

## 5. Discussion

In recent years, there has been a significant increase in the global demand for agricultural products, particularly grains. This trend is expected to continue due to the growing world population and changing lifestyles. Consequently, ensuring the safety of agricultural crops has become a global concern. Mycotoxins, among the most important contaminants in agriculture and food, have been linked to various harmful and irreversible effects on human and animal health, including carcinogenicity, genotoxicity, immunotoxicity, and mutagenicity.

Various tests have been conducted on different types of animal feed to assess mycotoxin contamination, each with its own set of advantages and disadvantages. Numerous screening methods, such as immunoassay-based techniques, biosensors, and non-invasive approaches, have been developed. However, to confirm positive findings, chromatographic methods, such as liquid chromatography (the most commonly used), gas chromatography, and thin-layer chromatography, are still employed. Table 6 lists various methods for screening mycotoxins in wheat.

This document examined the validation of the Myco 7 kit in accordance with Commission Decision 2002/657/EC (19). The results demonstrate that the kit is suitable for the simultaneous screening of 7 different mycotoxins in wheat flour samples within the validated ranges. The CCβ values were observed to be lower than the defined MRPLs set by the European Commission for all 7 mycotoxins. This method was observed to be fast, sensitive, and simple. All of the samples, whether packed or in bulk, were free from the screened mycotoxins. Freitas et al. obtained similar results, and the precision data they obtained are consistent with EU legislation performance criteria. Their results highlight that this method is a valuable and cost-effective screening method for the simultaneous semi-quantification of mycotoxins (24). Table 6 summarizes some examples of different mycotoxin screening methods.

**Table 6.** Assessment of Different Screening Methods of Mycotoxins in Wheat

Technique	Matrix	Advantage	Disadvantage	Ref
**Immunoassay-Based Methods: ELISA (enzyme-linked immunosorbent assay); LFIA (lateral flow immunoassay); - FPIA (fluorescence polarization immunoassay)**	A wide range of feed ingredients	Simple; Cheap; do not require sophisticated equipment or skilled personnel; -Require minimal or no sample pretreatment; -Portable	Can only detect a small group of mycotoxins; possibility of cross-reactivity with structural analogs; Quite time-consuming (ELISA)	(25, 26)
**Biosensors and biosensor-based methods: -Biochip array technology**	A wide range of feed ingredients	-Simple; Rapid; Cheap; do not require skilled personnel; require minimal or no sample pretreatment; -High-throughput (simultaneous determination)	Requires specific instrument; Possibility of cross-reactivity with structural analogs	(27-29)
**Noninvasive methods: Infrared spectroscopy (NIR); Raman spectroscopy (RS)**	A wide range of feed ingredients	Simple; rapid; in situ analysis; environmentally friendly; require slight or no sample preparation;	Limited application due to:; lack of appropriate calibration materials and; high matrix dependency; require skilled personnel; only useful at high contamination levels; require modern chemometrics methods	(25, 26)

Overall, as it is mentioned in Table 6, the advantages of the validated method outweigh other methods, and its disadvantages can be neglected compared to others.

**Figure 2.**
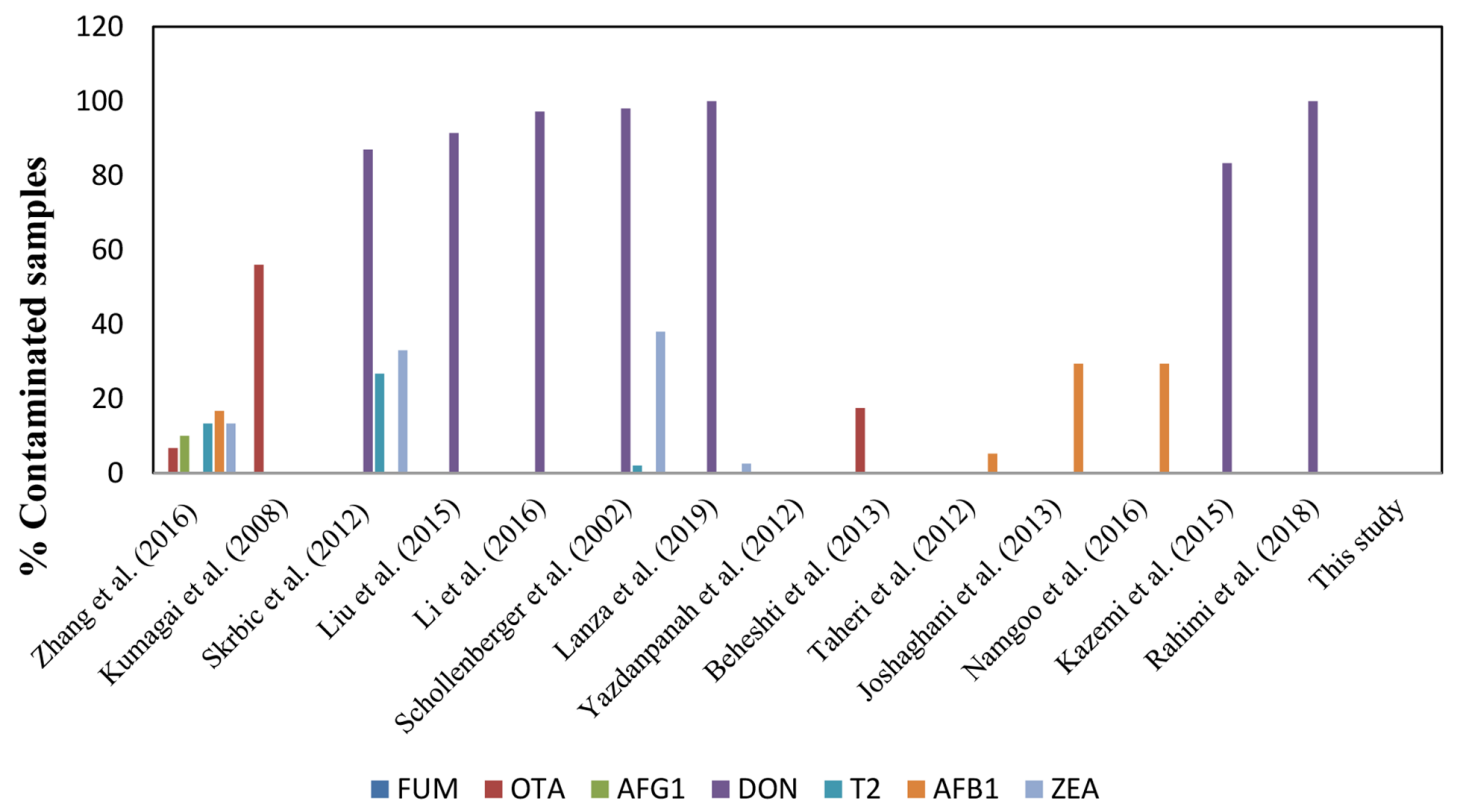
Incidence of Mycotoxins in Wheat Flour Samples in Different Studies (30-43). (Abbreviations: FUM, fumonisin B1; OTA, ochratoxin A; AFG1, aflatoxin G1; DON, deoxynivalenol; T2, T-2 toxin; AFB1, aflatoxin B1; ZEA, zearalenone)

As shown in Figure 2, numerous studies on detecting mycotoxin contamination in wheat samples have been carried out worldwide using different techniques. In a study to detect 12 mycotoxin contaminations in wheat flour samples in Hungary, only 4 out of 33 samples were positive (44). The analysis of 30 wheat flour samples collected across China revealed OTA (6.7%), ZEN (13.3%), AFB1 (16.7%), AFG1 (10.0%), AFB2 (16.7%), AFG2 (3.3%) and T-2 (13.3%) in samples using LC-MS/MS over a multi-antibody immunoaffinity column (30).

However, Kumagai et al. analyzed 50 wheat flour samples from Japan using HPLC or LC/MS for the detection of aflatoxins, ochratoxin A, and fumonisins. They observed that 56% of the samples were contaminated with ochratoxin A (31). Several studies identified deoxynivalenol as the most frequently detected mycotoxin in wheat flour samples. An assessment of wheat flour samples collected in Serbia for 11 principal mycotoxin contaminations showed that 13 out of 15 samples were contaminated with deoxynivalenol, 5 with zearalenone, and 4 with T-2 toxin (36). In a study conducted in the Chinese province of Hebei, 11 mycotoxins in 348 wheat flour samples were analyzed by LC/MS/MS, revealing that 91.4% of the samples were contaminated with deoxynivalenol (37). Another study in China evaluated 359 wheat flour samples collected in Shandong province using a multi-mycotoxin method based on ultra-high isotope dilution. They showed that 97.2% of the samples tested positive for deoxynivalenol (35). In southwestern Germany, a study analyzed 60 wheat flour samples for mycotoxin contamination using HPLC or GC-MS. Among the total samples, 98% were contaminated with deoxynivalenol, 2% with T-2 toxin, and 38% with zearalenone (38).

On another continent, Dos Santos et al. conducted an analysis of 200 wheat flour samples from southern Brazil using ultra-performance liquid chromatography-mass spectrometry (UPLC-MS/MS) to detect 12 regulatory and non-regulated mycotoxins. They demonstrated that all samples were contaminated with 2 to 3 mycotoxins, mainly zearalenone, deoxynivalenol, and T-2 toxin (44). Similarly, an analysis of 39 wheat flour samples collected throughout the northern region of Rio Grande do Sul state in Brazil revealed 39 positive samples for deoxynivalenol and 1 sample for zearalenone contamination. This analysis was conducted using a QuEChERS method and UPLC-MS/MS analysis (39).

In Iran, a survey reported that there was no zearalenone contamination in 18 wheat flour samples collected from the Tehran retail market and analyzed by HPLC (40). In a study in Khorasan province, ochratoxin A was detected in 17.5% of 40 wheat flour samples after HPLC analysis (41). In another study, 200 flour samples collected from Golestan province were assessed with the HPLC method with immune-affinity chromatography; only 3.1% and 7.4% of the samples were positive for aflatoxin B1 in summer and winter, respectively (42). Another study was conducted in the same province, where 29.4% of the wheat samples showed traces of aflatoxin, although none was above the standard value (43). A third study in Golestan province showed that 29.4% of samples were contaminated; however, only the concentration of aflatoxin B1 was above permitted levels in one of them (32).

In one study, 96 wheat flour samples collected in Guilan province were tested for deoxynivalenol contamination by enzyme-linked immunosorbent assay, and 80 samples (83.3%) were positive (33). The analysis of 150 wheat flour samples collected in Kermanshah, the western part of Iran, showed that all samples were contaminated with deoxynivalenol and deoxynivalenol-3-glucoside using the HPLC method (34).

The present study revealed that 39 bulk and packaged wheat flour samples were compliant by screening with the Myco 7 array kit. As shown in Figure 2, there are variations between the results of previous studies with each other and with the results of this study. Because mycotoxins are natural food contaminants, and mycotoxin production is related to the humid subtropical climate and storage conditions during the wheat-growing season, humidity during the wheat-growing season and storage conditions could be the reason for different concentrations of mycotoxins in various studies. Therefore, special attention should be dedicated to mycotoxin contamination in foods in different seasons and storage conditions.

To the best of our knowledge, no other similar study that screened 7 various mycotoxins in wheat flour samples using the mentioned technology has been carried out in Iran. The variety of methods and numerous studies on the detection of mycotoxin contamination in cereals indicate the global importance of this topic. Due to the significance and urgency of this issue, it is recommended that a more extensive survey with a diverse range of samples from all over the country be conducted.

### 5.1. Conclusions

This is the first study presenting the validation of Myco 7 array technology in wheat flour samples according to Commission Decision 2002/657/EC in Iran. Mycotoxin contamination in grains can lead to significant economic losses for farmers and pose serious health risks. The validated method meets the need for rapid and simultaneous screening of seven mycotoxins in various wheat flour samples with simple sample preparation. The test process of the Myco 7 kit was easy to conduct, and the results produced were straightforward to interpret.

Despite the results showing no mycotoxin contamination in wheat flour samples, due to the potential health and economic consequences, it seems necessary to regularly monitor for high-prevalence mycotoxins in different types of packaged and bulk wheat flour samples across various seasons and environments and other grain types. Therefore, it is imperative to conduct further research to monitor mycotoxins in various food items and estimate the average dietary exposure and health risk assessment of mycotoxins for key foods in Iran.
